# Hepatitis C Virus NS5B Sequence-Based Genotyping Analysis of Patients From the Sharkia Governorate, Egypt

**DOI:** 10.5812/hepatmon.12706

**Published:** 2013-12-17

**Authors:** Ahmed Elsadek Fakhr, Mahmoud Reza Pourkarim, Piet Maes, Amal Hassan Atta, Ayman Marei, Magda Azab, Marc Van Ranst

**Affiliations:** 1 Laboratory of Clinical and Epidemiological Virology, Rega Institute for Medical Research, Department of Microbiology and Immunology, KU Leuven, Leuven, Belgium; 2 Microbiology Department, Faculty of Medicine, Zagazig University, Zagazig, Egypt; 3 Blood Transfusion Research Center, High Institute for Research and Education in Transfusion Medicine, Tehran, IR Iran

**Keywords:** Hepatits C Virus, Genotype, Egypt, Multilocus Sequence Typing

## Abstract

**Background:**

Chronic hepatitis C virus infection and its sequela are major health problems facing the Egyptian community. The high prevalence and spread rates of the disease require serious actions to stop or decrease these rates. Determination of HCV genotypes and subgenotypes adds significant knowledge about the epidemiology of the disease, and provides an added value in the decision making process of what strategy to follow and what therapy response to expect. The molecular epidemiology and genetic variability of HCV variants circulating in Egypt still need further analysis.

**Objectives:**

The study was held to evaluate the genotype and subgenotype of the hepatitis c virus circulating in Sharkia as one of the large governorates of Egypt, which was not included in any study for genotyping of the virus before.

**Patients and Methods:**

The HCV molecular epidemiology in Sharkia governorate was studied using direct sequencing and further phylogenetic analysis of a partial NS5B region of the HCV genome from 63 patients. HCV genotype and subtype were successfully determined in 62 out of 63 patients.

**Results:**

The highest prevalent genotype was genotype 4a, which was found in 57 patients (92%) followed by 2 isolates (3%) with genotype 4o, 2 strains (3%) with genotype 1g and one isolate (2%) with genotype 4n.

**Conclusions:**

This molecular epidemiology study revealed high prevalence of HCV genotype 4, subtype 4a among Egyptian patients residing in Sharkia governorate, Egypt.

## 1. Background

Hepatitis C virus (HCV) infectionis estimated to affect 130–170 million people worldwide (1). Although the clinical manifestation of HCV acute infection is generally mild or asymptomatic, it can lead to chronic liver disease, cirrhosis, and hepatocellular carcinoma (HCC). The main indication for liver transplantation in the industrialized world is related to HCV infection (2).

HCV is a positive-sensed single-stranded RNA virus belonging to the Flaviviridae family. The HCV genome is an RNA molecule of approximately 9600 nucleotides, structured in a coding region that contains one large open reading frame (ORF), flanked by non-translated regions (NTR) at the 5’ and 3’ ends encoding a polyprotein precursor of about 3,000 amino acids. The precursor is cleaved into at least 10 different proteins comprising the structural proteins, core, E1, E2, and p7 as well as the non-structural proteins, NS2, NS3, NS4A, NS4B, NS5A, and NS5B (3). HCV has been classified into 6 major genotypes and numerous subtypes (4). 

HCV genotype determination is now an important practice before patient management. In addition, it is a useful means for better understanding of epidemiological and virological features of this virus. Many genotyping methods targeting different regions of the HCV genome have been developed. Sequencing of a genome region divergent enough to discriminate types and subtypes is considered to be the most accurate method. Direct sequencing of HCV NS5B, core, and envelope regions has proven to be reliable for classification of HCV to different genotypes (5-7).

The prevalence of HCV infection in Egypt is the highest all over the world. Previous studies revealed that most of Egyptian patients are infected with genotype 4 with a predominance of subtype 4a (8). According to epidemiological and molecular evolutionary analysis on HCV isolates from Egypt, the HCV genotype 4 epidemic was linked to the campaigns of parenteral administration of antischistosomal treatment, which were held till the mid- 1980s (9, 10). However, the persistence of high rates of infections even after afore mentioned treatment campaign was stopped is attributed to many other risk factors including prevailing social and cultural behaviors. At present, it is assumed that the major route of transmission is health-care related measures using inadequately sterilized devices, in addition to some procedures done by non-medical personnel and traditional practitioners (8, 11). Transmission among persons of the same family and sexual transmissions also take part in the maintenance of these high prevalence rates (12). 

**Figure 1.**
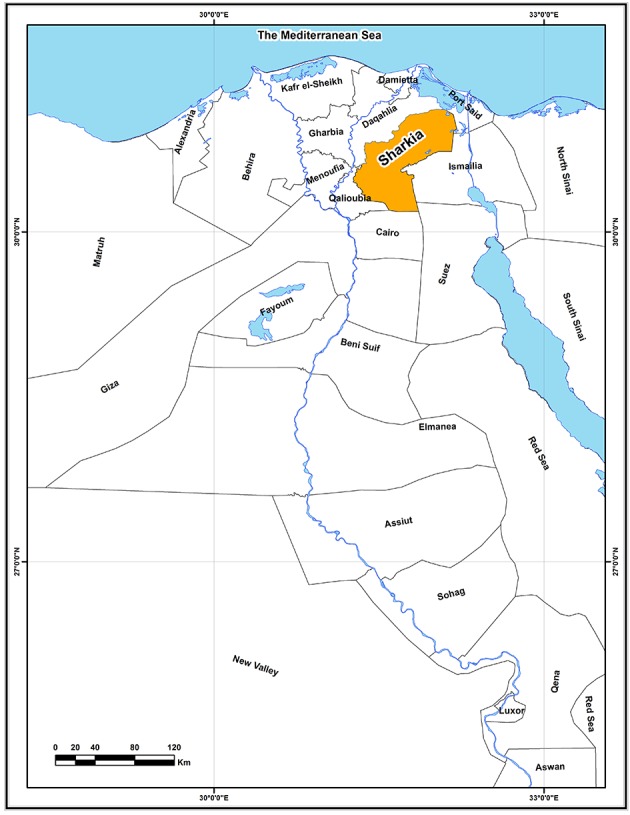
Location of Sharkia Within Egypt Governorates

In contrast to its high prevalence, epidemiological data on the subtypes of HCV in Egypt are still lacking. In this article we report the prevalence of HCV genotypes and subtypes in 63 HCV-positive patients whom were candidate to antiviral therapy provided by a Central Referral Hospital in Sharkia. The Sharkia governorate, also spelled Ash-Sharqīyah, is located on the eastern part of the Delta governorates; to the east of the Northern part of the River Nile (Figure 1).Sharkia is the second after Cairo governorate in terms of number of population. Similar to the situation nationwide, few data are known about HCV genetic variability in this region. 

## 2. Objectives

The main objective of this investigation is to investigate the genotype and subgenotype distribution of HCV in the Sharkia governorate.

## 3. Patients and Methods

### 3.1. Study Population

This study was implemented on 63 HCV RNA positive patients candidate to therapy from the Sharkia governorate, Egypt. Patients consisted of 48 male’s and15 females, with an age ranging from 20 to 61 years old. The samples were provided bya Central Referral Hospital in Sharkia.

### 3.2. HCV RNA Extraction and RT-PCR

HCV-RNA extraction was carried out by using the QIAamp® Viral RNA Kit (Qiagen/Westburg, The Netherlands), following the manufacturer’s instructions.

HCV genotypes were identified via direct sequencing of non-structural (NS5B) viral genes with universal primers described by Murphy et al. 2007 (7). RT-PCR was done using a one-step RT-PCR kit (QIAGEN, Inc.). The primers that were used in this study are sense 5’-TTCTCRTATGAYACCCGCTGYTTTGA-3’ and antisense 5’-TACCTVGTCATAGCCTCCGTGAA-3’.

### 3.3. DNA Purification and Sequencing

The PCR amplicons were purified by using innuPREPPCRpure kit (Analytik Jena, Germany), and sequenced using the dideoxynucleotide chain termination method with the ABI PRISM®BigDye Terminator Cycle Sequencing Reaction kit (Applied Biosystems, Foster City, CA, USA) Sequence analysis was performed with the ABITM3130 Genetic Analyzer (Applied Biosystems, Foster City, CA, USA). The sense and antisense primers described above were used as sequencing primers. The chromatogram sequencing files were inspected using Chromas 2.3 (Technelysium, Helensvale, Australia). The genotype of each sample was determined by comparing its sequence with those of HCV prototypes obtained from GenBank, followed by further genetic analysis. The DNA alignments were generated with ClustalX version2.0 software (13). Bootstrap analysis and phylogenetic tree were determined with the MEGA software version 4.0.1 by using the neighbor-joining method (14).HCV genotypes were classified according to the nomenclature proposed previously by Simmonds et al. (2005) (4). Fifty-six reference sequences for HCV genotypes and subtypes were obtained from GenBank and used for constructing phylogenetic trees. 

## 4. Results

The NS5B region corresponds to a 389 nt fragment covering the middle section of the NS5B gene of the HCV genome. The direct sequencing and phylogenetic analysis of the HCV NS5B gene was successful in 62out of 63 positive samples. Samples number Eg 21 and Eg 22 had been sequenced two times and both of them have exactly the same sequence in each run. They were expressed in the tree as one sample (Eg 21). The result of the study revealed that subtype 4a was the most prevalent in this population with a total number of 57 (92%).Subtype 1g and 4o were each identified in 2 samples (3%), and 4n was identified in 1 sample only (2%). 

**Figure 2.**
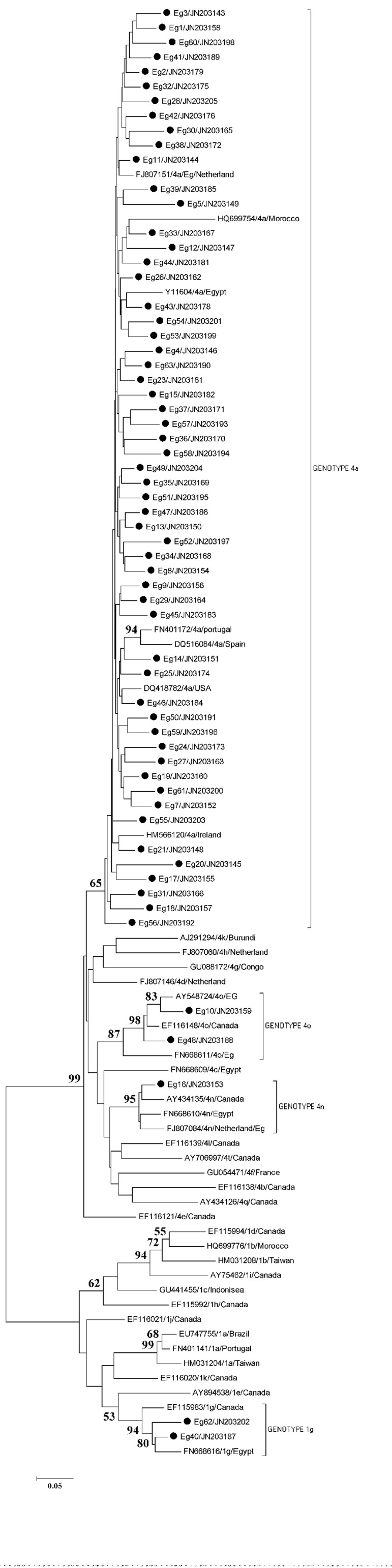
Neighbour-Joining Phylogenetic Tree Based on NS5B Gene Analysis From 62 Egyptian Strains, 37 Reference Strains With Different Genotypes Retrieved From GenBank The reference sequences were named after their accession numbers/subgenotype/country of origin. The study strains were marked by the black dot. The numbers at the nodes represent the percentages of bootstrap support values (percentages obtained for 1000 replicates).

Preliminary phylogenetic analysis containing 56 sequences (data not shown) was performed and allowed a selection of 37 additional GenBank sequences corresponding to the most related ones to the study isolates. These reference sequences were named after their accession numbers, subgenotype and country of origin. Partial NS5B coding sequences obtained from Egyptian samples were aligned with the reference sequences. The phylogenetic tree obtained by performing neighbor-joining (NJ) analysis of the alignment of sequences is shown in Figure 2. Fifty-six of the isolates cluster together with the reference sequences of genotype 4a. Low heterogeneity was observed between the studied sequences and the reference sequences from different countries of the same subgenotype. 

Three samples clustered in a different branch with reference sequences from genotype 4. HCV isolates Eg-10 and Eg-48 clustered with reference sequences of HCV genotype 4o from Egypt and from Canada with a high bootstrap value of 98%. The Eg-16 isolate clustered with three reference sequences of genotype 4n from Egypt, Canada and an Egyptian immigrant from the Netherlands, with a bootstrap value of 95%.Another 2 samples (Eg-40 and Eg-62) branched with reference sequences of HCV genotype 1g from Canada and from Egypt with a bootstrap value of 94%.Sequences were submitted to GenBank (accession numbers: JN203143 to JN203205).

## 5. Discussion

According to different reports, the prevalence of HCV in Egypt ranges from 11% to higher than 14% of the general population, 5–7 million people with active infection having detectable HCV RNA and over 500,000 new infections each year (15-17). In comparison to its high prevalence, which is equal to 10-20 folds that of the USA (18); few studies were carried out to investigate the HCV genotype and subgenotype distribution in the different geographical areas in Egypt?

It is known that genotype and even subtypes differences of HCV havea high impact on interferon responsiveness and give an idea about the natural history of the disease (19). In Egypt, the unique epidemic of HCV genotype 4 occurred in conjunction with the parenteral anti-schistosomial campaigns, which began during the 1940s and stopped in 1980, when oral therapy was, introduced (20). Previous studies demonstrated a low level of heterogeneity among HCV genotypes isolated from Egypt, especially in isolates from the Nile Delta and the Nile Valley, where parenteral anti-schistosomial campaigns were predominantly held. Ray and coworkers showed by one hundred ninety serum specimens, obtained from subjects in 15 geographically diverse governorates (Sharkia was not included),that the Egyptian HCV epidemic is composed of multiple lineages of genotypes 1 and 4, including subgenotypes 4a, 4o and 1g (18). In Cairo, Abdel-Hamid and his colleagues stated that of 131 successfully analyzed samples, 83 were of subtype 4a, but five other subtypes within genotype 4 were also observed, as well as three genotype 1b, five genotype 1g and one genotype 3a samples (21). 

In a study on 206 samples collected from the Kasr El-Aini School of Medicine and the National Cancer Institute, Cairo, Egypt, HCV genotype 4 was detected in 186 samples (90.3%). Five other subtypes could be also identified: subtypes 4a, 4d, 4m, 4n, and 4o. They also found that 9.7% of the patient’swereinfected with HCV genotype 1g and 1a (22). In the same study, a distribution map of HCV genotypes dividing Egypt into five zones, including 17 governorates, was created: North Central Egypt, Central Egypt, South Central Egypt, South Egypt and West Egypt (also in this study Sharkia was not included). No significant geographic pattern to the distribution of HCV genotypes, either by the subjects’ present place of residence or their place of origin, was detected. 

Another study, from the Alexandria district, showed that 78% of the isolates were HCV genotype 4a variants, whereas the remaining identified variants were 4m (11%), 4o (5.5%), 4n (2.7%) and 4p (2.7%) (23). In Ismailia, using direct sequencing and phylogenetic analysis of the HCV NS5B gene, 15 cases (78.9%) were subtype 4a, two(10.5%) were subtype 1g, and one (5.2%) was related to subtype 4o (24).

In conclusion, this study shows that HCV genotype 4ais predominant in the governorate of Sharkia, and it confirms that the Egyptian HCV epidemic is composed of multiple lineages of genotypes 4 and 1.
